# Supplementary material for: Direct observation of photoinduced carrier blocking in mixed-dimensional 2D/3D perovskites and the origin
Source: Nat Commun. 2022 Oct 20;13:6229. doi: 10.1038/s41467-022-33752-5 (PMC9585031; doi:10.1038/s41467-022-33752-5)
Supplement: Supplementary file 1 — Supplementary Information [file 41467_2022_33752_MOESM1_ESM.pdf]

Supplementary Information

Direct Observation of Photoinduced Carrier Blocking in Mixed-Dimensional 2D/3D Perovskites and the Origin

Yu et al.

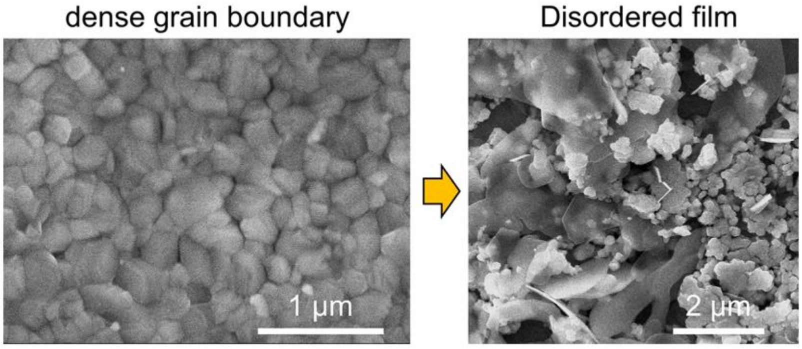

Supplementary Figure 1. Failure to obtain the GIHP film starting from a common $MAPbI_3$ film. The SEM image of the resultant HP film (right panel) by treating a common $MAPbI_3$ film (left panel, using diethyl ether rather than toluene as the antisolvent) with PEA^+.

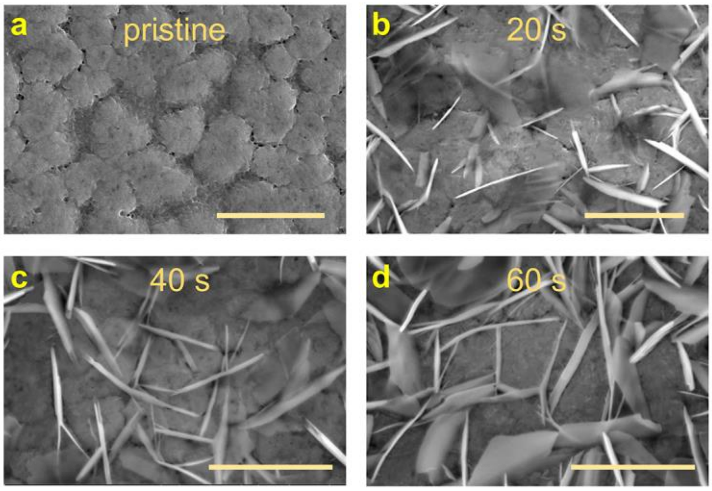

Supplementary Figure 2. Evolution of the GIHP film during treatment. (a)-(d) SEM micrographs of the GIHP film after different treating times. The scale bars in a-d are 5 μm.

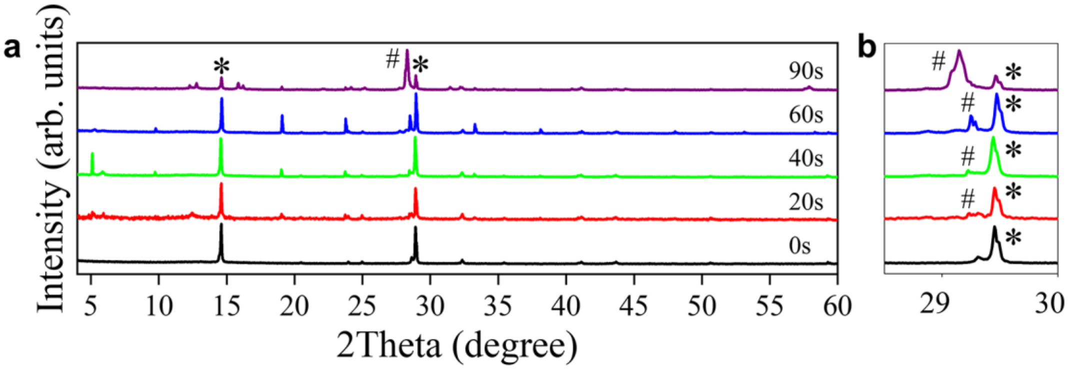

Supplementary Figure 3. Evolution of the XRD pattern during treatment. (a) The treating time-dependent XRD pattern of the GIHP film. # denotes the diffraction peak of 2D HPs, while * denotes the diffraction peaks of the parent 3D $MAPbI_3$. (b) A zoomed view that shows the growing diffraction intensity of 2D HPs and the concurrent waning diffraction intensity of the 3D HPs upon increasing treating time. Besides, the 2D HP diffraction peak shifts towards the smaller angle region, indicating the decrease of the average *n* value (quantum well width) that reduces the interlayer distance.[1]

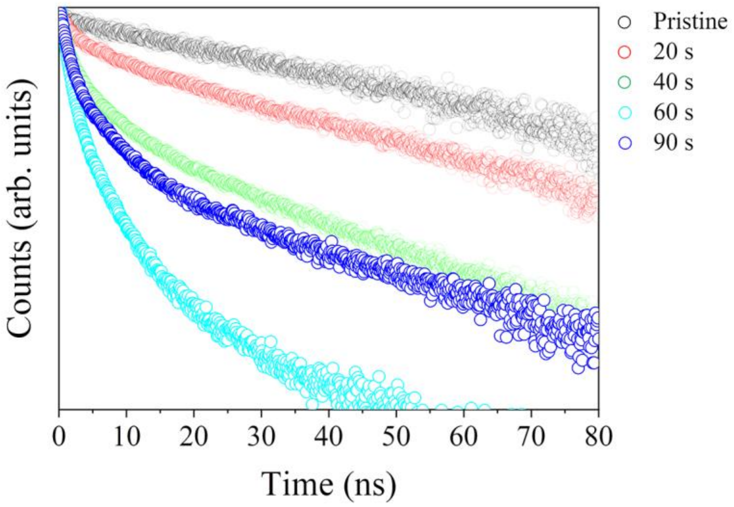

Supplementary Figure 4. The transient PL spectrum characterization. The transient PL spectra of the GIHP film with different treating times.

Supplementary Note 1: A long-pass filter of 532 nm was used so that the photon counter counts the photons from all the HP phases in the film. The overall PL lifetime decreases initially due to the formation of the dimensionality-reduced 2D HP crystallites that inherently own higher recombination rates, so that the passivation effect is averaged out. But the PL lifetime does not constantly decrease. It can be observed that the PL lifetime of the 90 s-treated sample increases compared to the 60 s-treated sample, which unambiguously confirms the passivation effect.

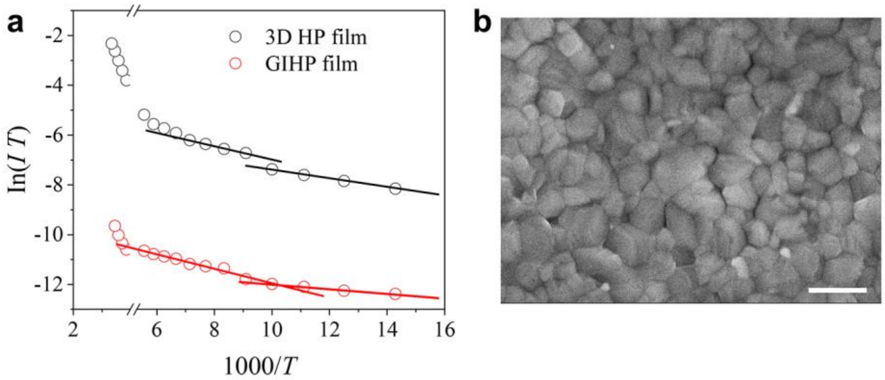

Supplementary Figure 5. Characterization of the activation energy of carriers. (a) The temperature-dependent conductivity of a typical 3D $MAPbI_3$ film and the GIHP film. (b) The SEM micrograph of the 3D $MAPbI_3$ film. The scale bar is 500 nm.

Supplementary Note 2: The activation energy (E_a) of carriers was extracted from the temperature-dependent conductivity curve according to the Nernst-Einstein relation:

$$\sigma(T) = \frac{\sigma_0}{T} \exp\left(-\frac{E_a}{kT}\right) \quad (1)$$

where σ is the conductivity; k is the Boltzmann constant; σ_0 is a constant; T is temperature. Assuming an unchanged shape of the films when varying the temperature, σ can be replaced with electricity (denoted as I). The activation energy of carriers is extracted from the low-temperature realm, while the high-temperature realm corresponds to the activation energy of ion migration.[2] The carrier activation energies of the $MAPbI_3$ film are calculated to be 23 meV and 15 meV, and the values are 22 meV and 8 meV for the GIHP film.

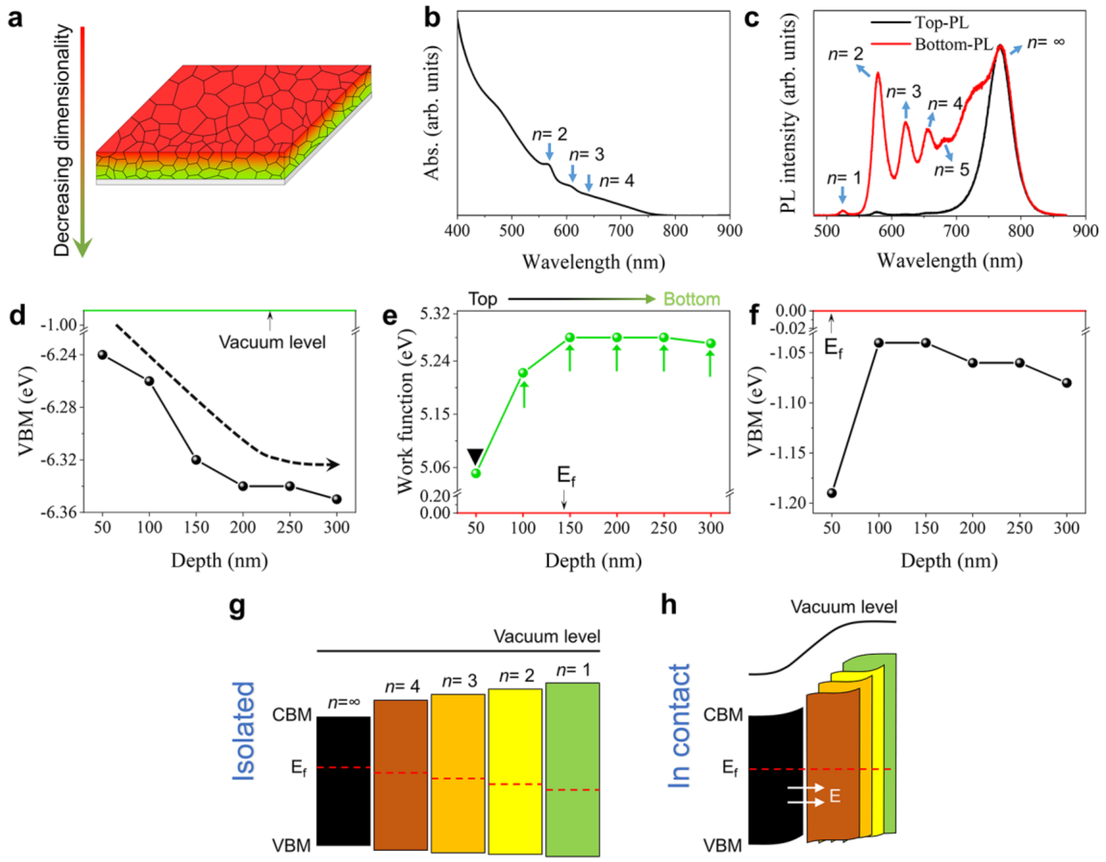

Supplementary Figure 6. Measuring the built-in potential of the 2D/3D HP interface via the depth-profile UPS measurement. (a) Schematic illustration of the axial dimensionality transition in a spin-coated $PEA_2MA_4Pb_5I_{16}$ film. (b) The corresponding absorption spectrum showing characteristic peaks of dissimilar phases. (c) Corresponding PL spectra probing at the top (the HP side) and the bottom (the quartz side) of the film. (d) Depth-profile absolute VBM positions versus a fixed vacuum level. (e) Depth-profile work function versus the fixed Fermi level. (f) Depth-profile VBM positions versus the fixed Fermi level. Schematic illustrations of the energy band alignment of dissimilar 2D- and 3D HP phases (g) in a non-contacting situation (versus a fixed vacuum level) and (h) in a contacting situation (versus a fixed Fermi level). The arrows mark the direction of the built-in electric field.

Supplementary Note 3: In-situ UPS measurement in a phase-graded $PEA_2MA_4Pb_5I_{16}$ film (Supplementary Figure 6a) was conducted. The characteristic absorption peaks of a collection of dissimilar phases can be observed in the absorption spectrum (Supplementary Figure 6b), and the PL spectra (Supplementary Figure 6c) measured in the front and back surfaces suggest a 3D-to-2D gradient phase distribution, i.e., a decreasing average dimensionality, from top to bottom. Depth-profile UPS

measurement was then implemented to explore the electrostatic potential variation upon dimensionality transition.

The ionization potential increases with depth (Supplementary Figure 6d), meaning a progressively lowered absolute valence band maximum (VBM) position as the dimensionality decreases. Considering that the largest VBM offset of 0.15 eV is lower than the bandgap difference between 3D HP and 2D HPs (~1.6 eV for $n= \infty$, ~2.4 eV for $n= 1$, ~2.2 eV for $n= 2$, ~2 eV for $n= 3$), we reason a type-I band alignment between them in a non-contacting situation, and the CBM offset is higher than the VBM offset; On the other hand, the work function increases with depth (Supplementary Figure 6e), which implies a positive shift of the band (i.e. being p-type) upon decreasing dimensionality since the Fermi level is fixed during the measurement. Such band shift unveils the presence of a built-in potential between 2D- and 3D HP phases, and as a consequence, the initially lower absolute VBM positions of the 2D phases (in a non-contacting situation) are lifted to be above that of the 3D phase upon contact (Supplementary Figure 6f). Altogether, we can picture the energy band landscape as in Supplementary Figure 6g-6h. The detailed UPS data are shown in Supplementary Figure 7 below.

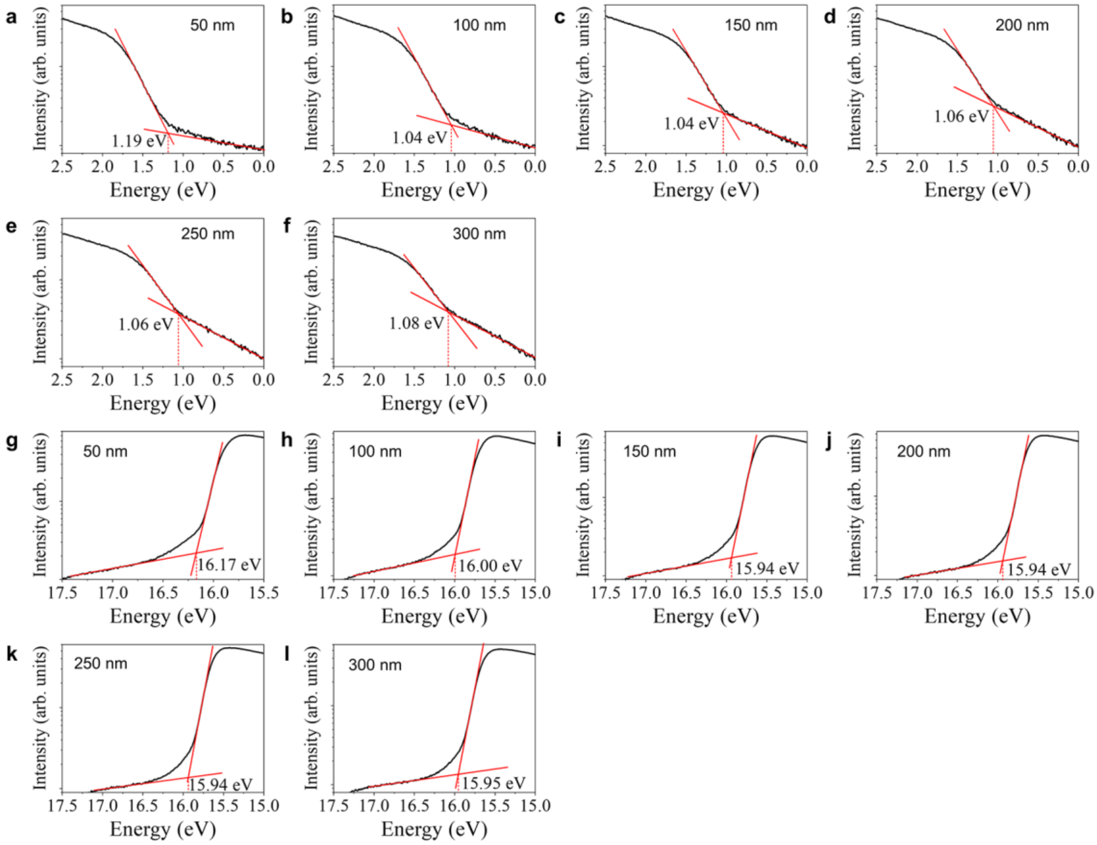

Supplementary Figure 7. Depth-profile UPS data of the phase-graded $PEA_2MA_4Pb_5I_{16}$ film. (a)-(f) The UPS secondary electron edge (SEE) data at different depths. (g)-(l) The UPS Fermi edge data at different depths. The etching depth was calibrated in a standard SiO_2 sample.

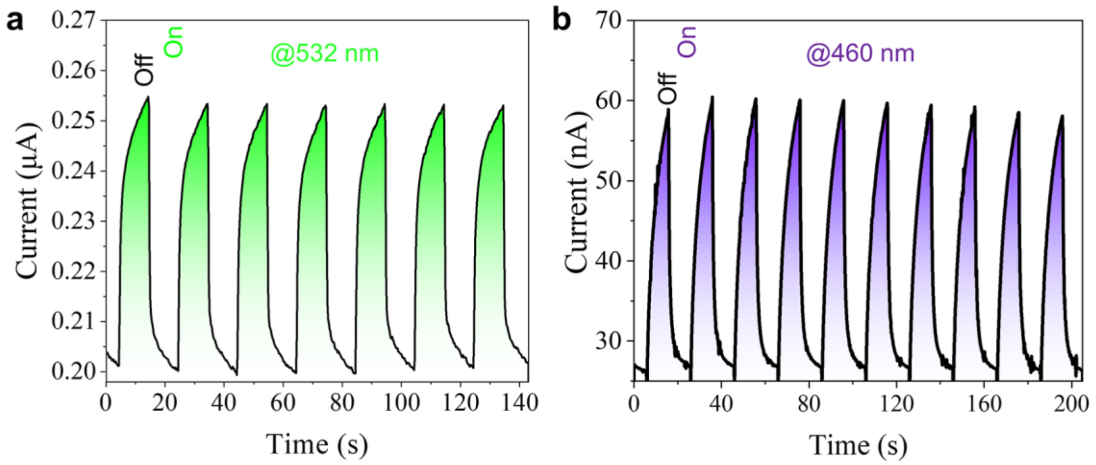

Supplementary Figure 8. *I-t* characteristics of the GIHP film. *I-t* curve of the GIHP film measured under periodic light-on and light-off states. The excitation wavelengths for (a) and (b) are 532 nm and 460 nm, respectively. The applied bias voltage is 0.5 V.

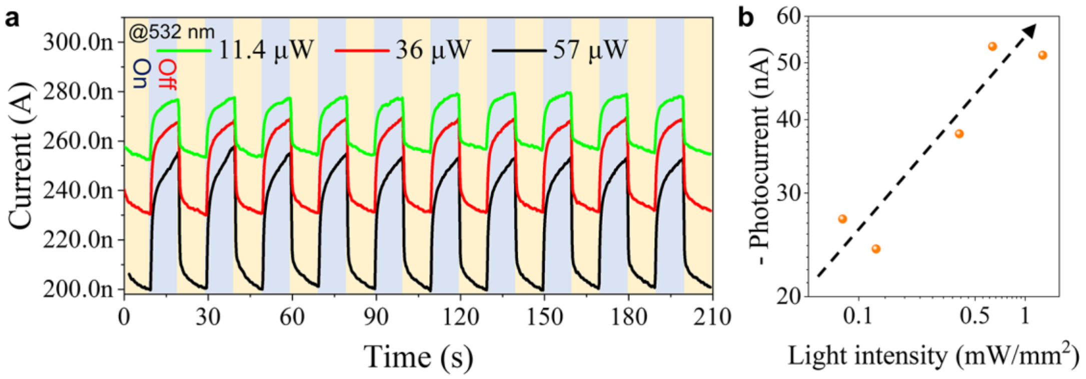

Supplementary Figure 9. Illumination intensity dependence of the photocurrent. (a) The *I*-*t* characteristics of the GIHP film under different excitation intensity levels (@532 nm). The applied bias voltage is 0.5 V. (b) The dependence of photocurrent on the illumination intensity.

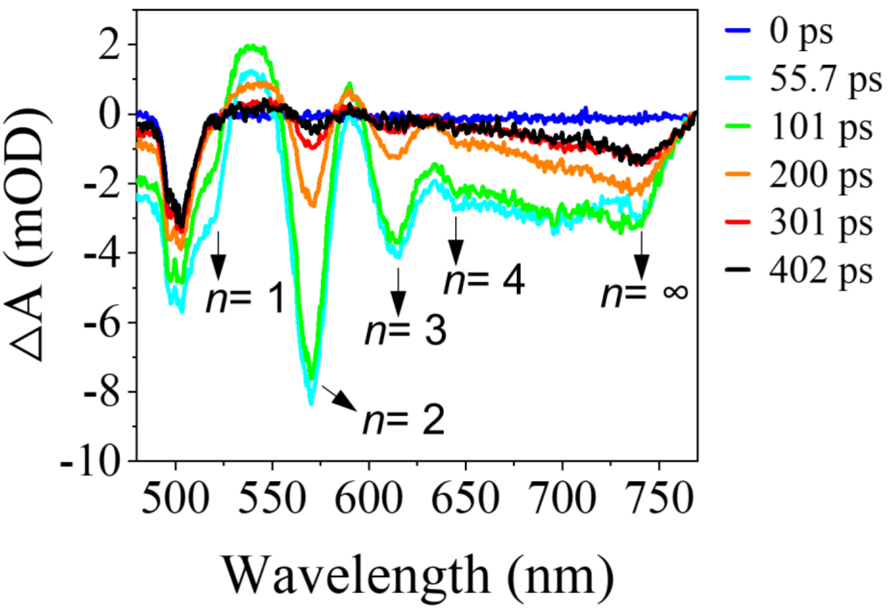

Supplementary Figure 10. TA spectrum characterization. TA spectra of the multiphasic $PEA_2MA_4Pb_5I_{16}$ film at different delay times.

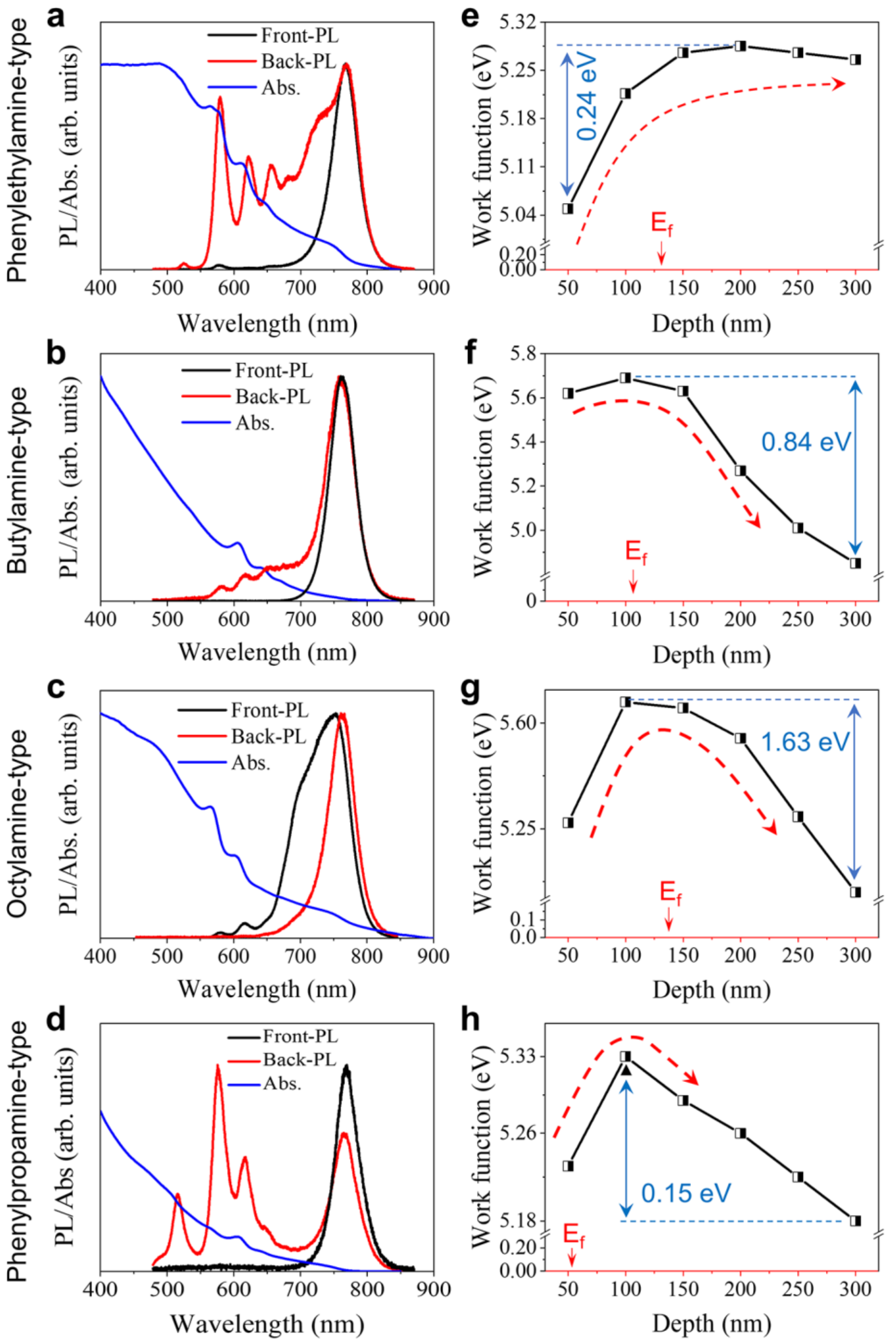

Supplementary Figure 11. Built-in potentials of different mixed-dimensional 2D/3D HP systems. (a)-(d) The front PL, back-PL, and absorption spectra of the PEA-type, BA-type, OA-type, and PPA-type phase-graded mixed-dimensional HP films (the nominal *n* values are all 5). (e)-(h) The depth-profile work function of these phase-graded films.

Supplementary Note 4: Four types of mixed-dimensional 2D/3D HP films with a gradient phase distribution (as indicated by the front-PL and back-PL spectra) were

fabricated for the depth-profile UPS measurement, and the largest work function offsets are adopted as the built-in potentials of the corresponding 2D/3D HP interfaces. The detailed UPS data are shown in Supplementary Figure 12-14.

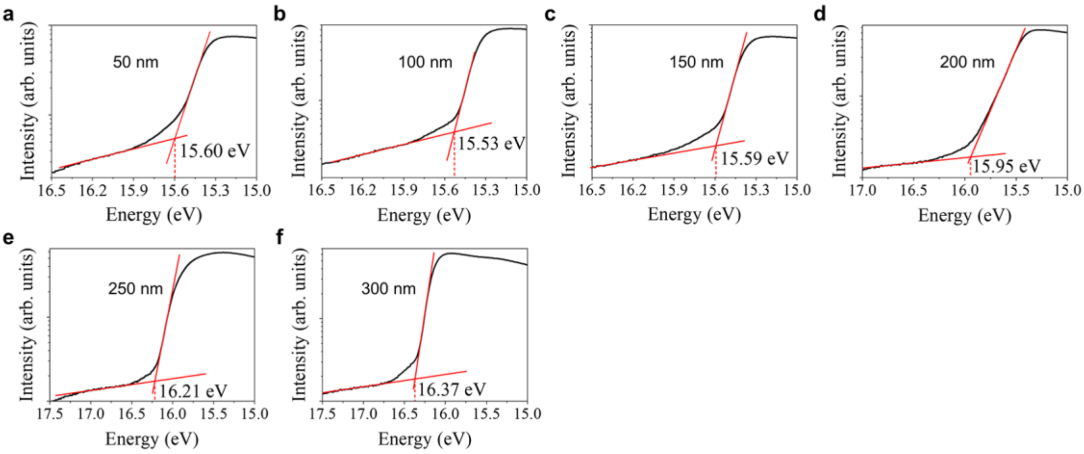

Supplementary Figure 12. UPS characterization of the $BA_2MA_4Pb_5I_{16}$ film. The UPS Fermi edge data at (a) 50 nm, (b) 100 nm, (c) 150 nm, (d) 200 nm, (e) 250 nm, (f) and 300 nm depths of the $BA_2MA_4Pb_5I_{16}$ film.

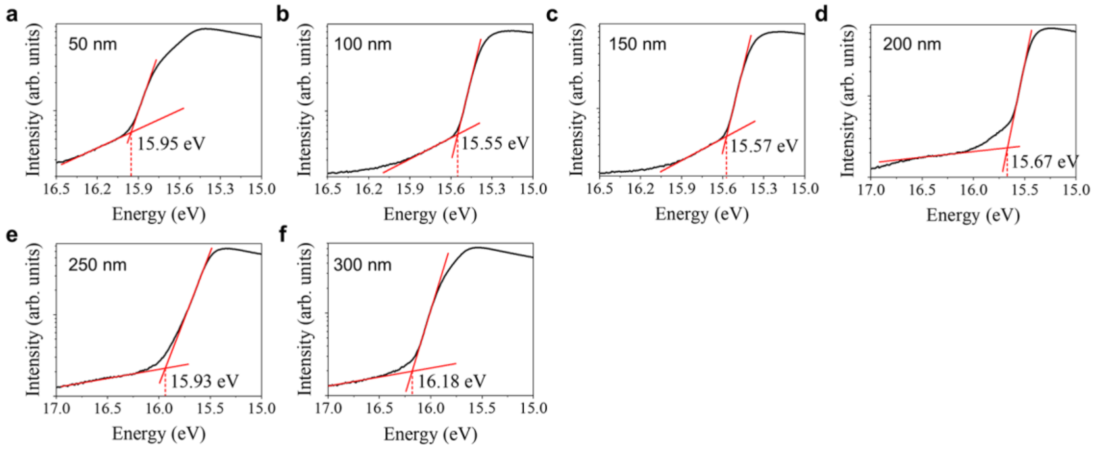

Supplementary Figure 13. UPS characterization of the $OA_2MA_4Pb_5I_{16}$ film. The UPS Fermi edge data at (a) 50 nm, (b) 100 nm, (c) 150 nm, (d) 200 nm, (e) 250 nm, (f) and 300 nm depths of the $OA_2MA_4Pb_5I_{16}$ film.

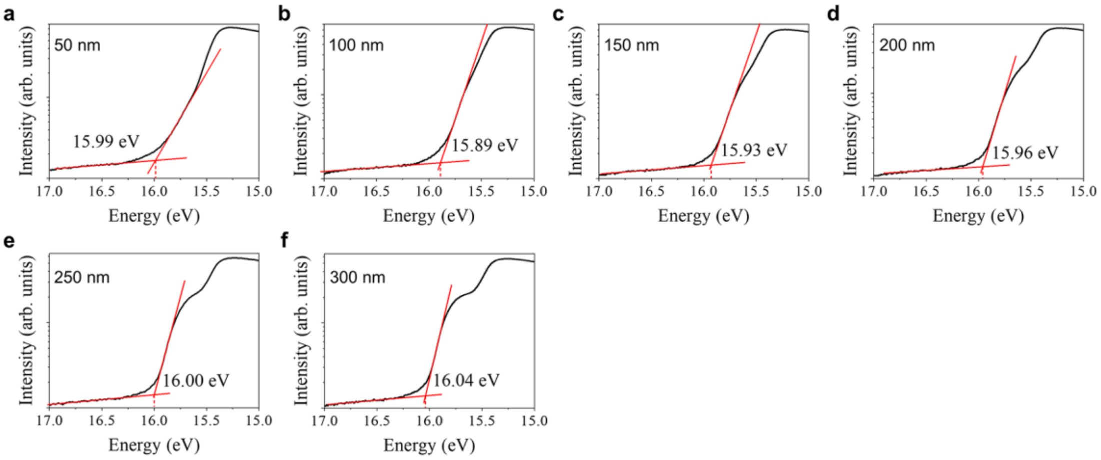

Supplementary Figure 14. UPS characterization of the $PPA_2MA_4Pb_5I_{16}$ film. The UPS Fermi edge data at (a) 50 nm, (b) 100 nm, (c) 150 nm, (d) 200 nm, (e) 250 nm, (f) and 300 nm depths of the $PPA_2MA_4Pb_5I_{16}$ film.

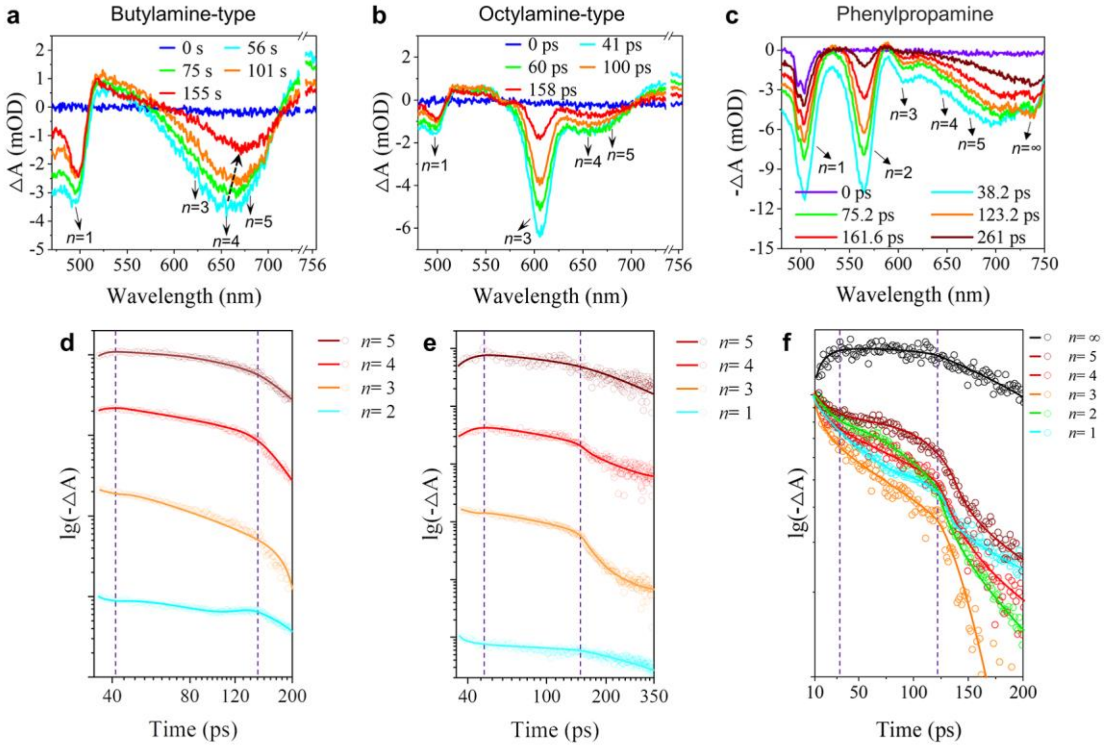

Supplementary Figure 15. TA characterization of different mixed-dimensional 2D/3D HP systems. (a)-(c) TA spectra at different delay times probed in the BA-type, OA-type, and PPA-type mixed-dimensional 2D/3D HPs; (d)-(f) TA kinetics of the 2D- and 3D HP phases probed at their characteristic wavelengths.

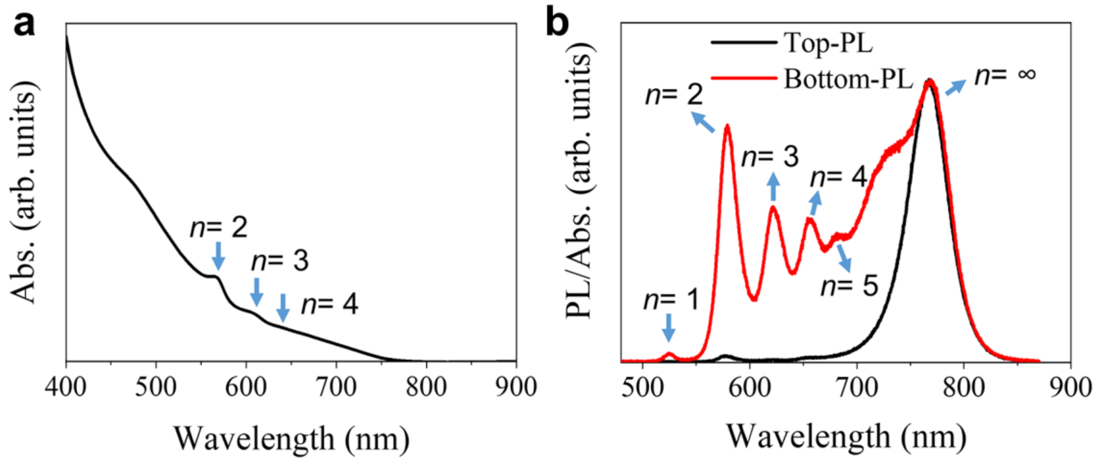

Supplementary Figure 16. The absorption spectrum and PL spectrum characterizations. (a) The absorption spectrum of the $PEA_2MA_4Pb_5I_{16}$ film for depth-profile phase arrangement measurement. (b) Corresponding PL spectra probing at the top (the HP side) and the bottom (the quartz side) of the film.

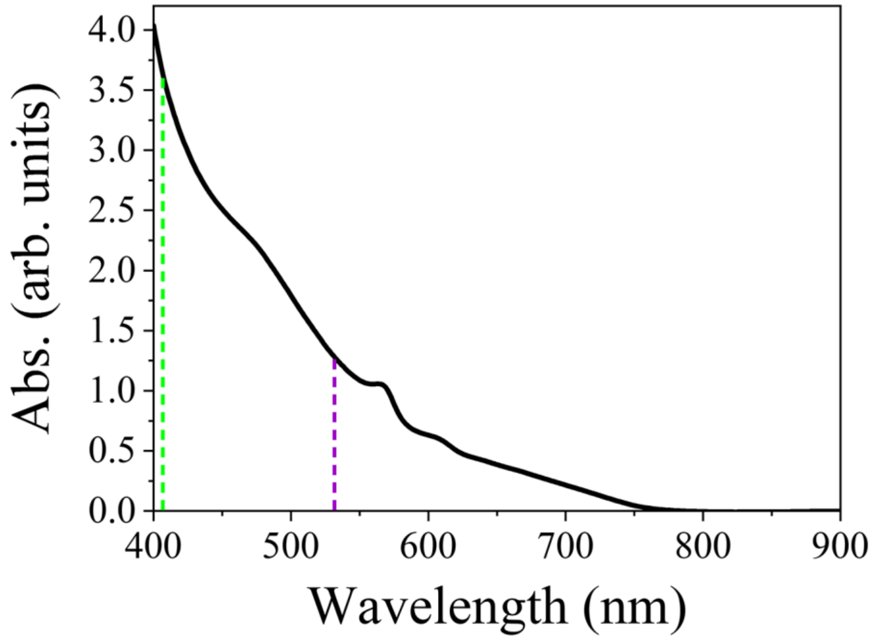

Supplementary Figure 17. The absorbance of the $PEA_2MA_4Pb_5I_{16}$ film at 405 nm and 532 nm. The absorption spectrum of the $PEA_2MA_4Pb_5I_{16}$ film for the one-photon PL mapping measurement. The green dashed line marks the absorption of the 532 nm excitation photon, and the blue dashed line marks the absorption of the 405 nm excitation photon.

Supplementary Note 5: The residual incident light intensity at a certain depth *z* can be obtained according to the Lambert-Beer law:

$$T(z)=T_0\exp(-\alpha\, z) \qquad (2)$$

where T_0 is the initial light intensity; α is the light absorption coefficient. The ordinate of the absorption spectrum A (absorbance) is defined as:

$$A=\lg(T_0/T_{th}) \qquad (3)$$

where T_{th} is the residual light intensity after penetrating the whole layer. Taking *z* as the layer thickness D, we can establish the following relationship by combining the above two formulas:

$$1/\alpha = D/(A \ln 10)$$

where $1/\alpha$ is the penetration depth. The A values for the 405 nm and 532 nm photons are 3.729 and 1.276, respectively. Therefore, we estimate their penetration depths to be 11.7% and 34.2 % of the layer thickness.

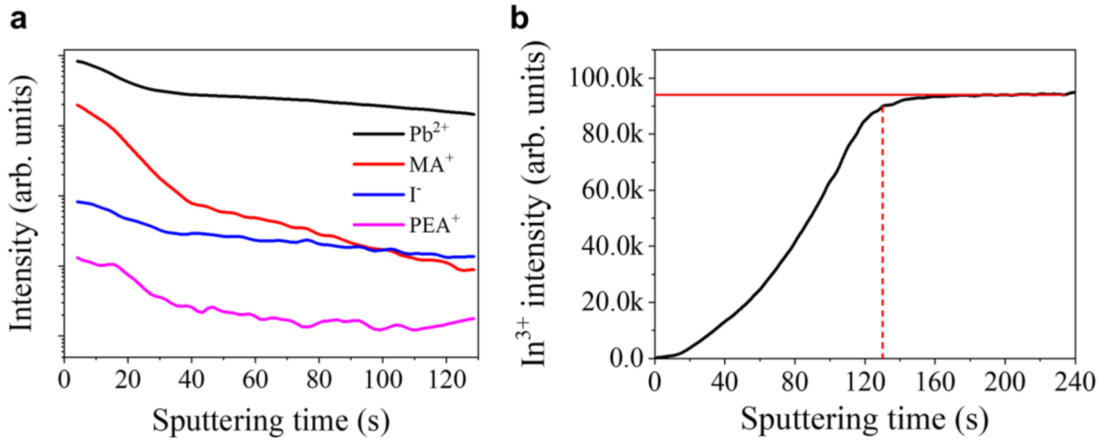

Supplementary Figure 18. TOF-SIMS characterization of the $PEA_2MA_4Pb_5I_{16}$ film. (a) Depth-profile ion distribution of the $PEA_2MA_4Pb_5I_{16}$ film. (b) The corresponding depth-profile intensity of In^{3+} (from the bottom ITO layer). We define the position with 95% of the peak intensity as the bottom of the film.

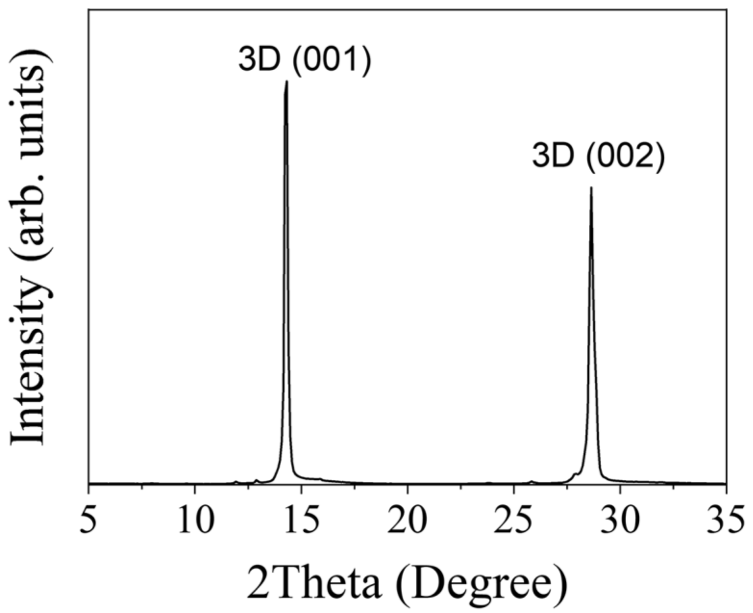

Supplementary Figure 19. One-dimensional XRD characterization. One-dimensional XRD pattern of the $PEA_2MA_4Pb_5I_{16}$ film. The XRD pattern shows a vertical orientation of the 2D fragments.

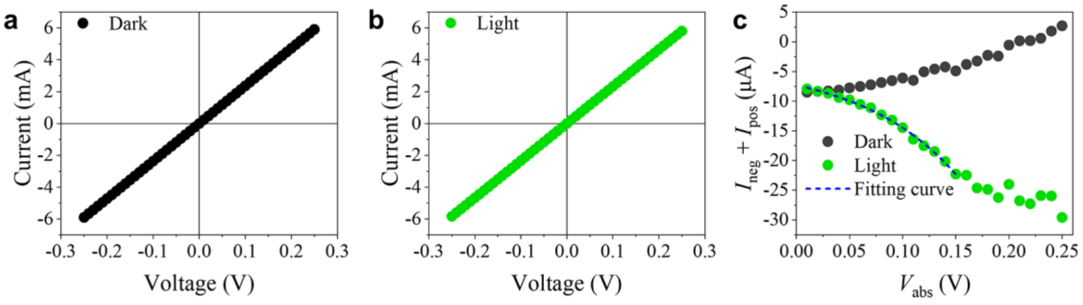

Supplementary Figure 20. In-plane electric characterization of the vertical ITO/$PEA_2MA_{2.67}Pb_{3.67}I_{12}$/Cu configuration. The second-cycle result of the vertical ITO/$PEA_2MA_{2.67}Pb_{3.67}I_{12}$/Cu to exclude experimental errors. The *I*-*V* characteristics measured (a) in darkness and (b) under one-sun illumination. (c) (I_{neg} + I_{pos}) versus V_{abs} characteristics in darkness and under illumination.

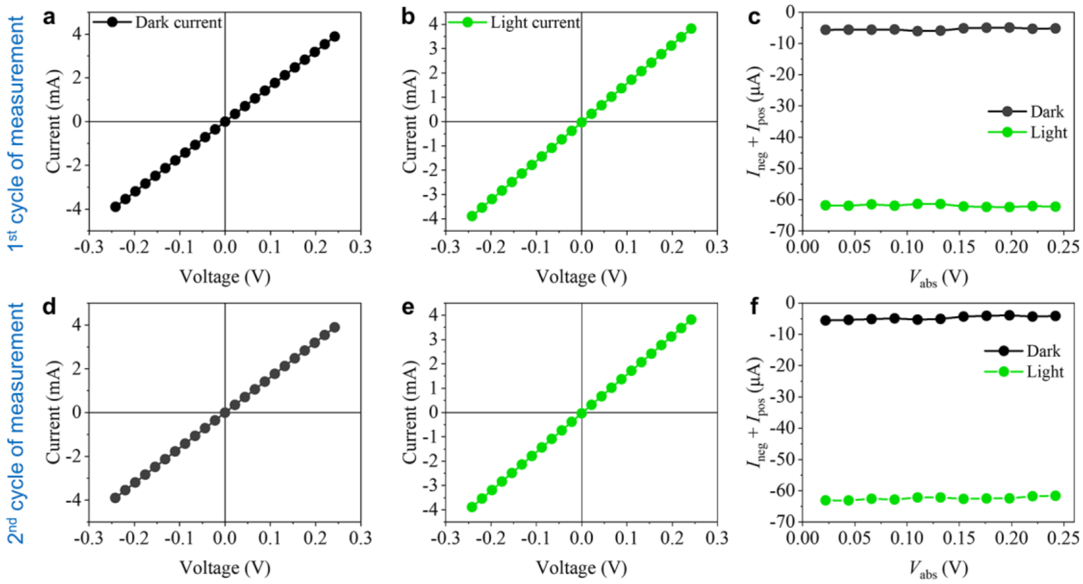

Supplementary Figure 21. In-plane electric characterization of the vertical ITO/$MAPbI_3$/Cu configuration. Two cycles of the *I*-*V* measurement of the vertical ITO/$MAPbI_3$/Cu. (a)-(c) are the first-cycle results, (d)-(f) are the second-cycle results. The *I*-*V* characteristics measured (a)&(d) in darkness and (b)&(e) under one-sun illumination. (c)&(f) (I_{neg} + I_{pos}) versus V_{abs} characteristics in darkness and under illumination.

Supplementary Note 6: For the vertical ITO/$MAPbI_3$/Cu configuration, the *I*-*V* characteristics measured in darkness (Supplementary Figure 21a) and under one-sun illumination (Supplementary Figure 21b) confirm an ohmic contact property. Supplementary Figure 21c shows the (I_{neg}+I_{pos}) versus V_{abs} characteristic in darkness and under one-sun illumination. It could be found that I_{neg} outcompetes I_{pos} across the sweeping V range, which indicates asymmetric electron transport that could be ascribed to the slight work function difference between ITO and Cu. Importantly, the same asymmetry remains when illuminated, and the higher current difference is due to photogenerated electrons. This result suggests an unchanged energy landscape of the ITO/HP/Cu configuration when illuminated, not only the energy landscape of the metal/semiconductor contacts but also that across the $MAPbI_3$ film. A repeated measurement presents the same result, which excludes experimental errors (Supplementary Figure 21d-21f).

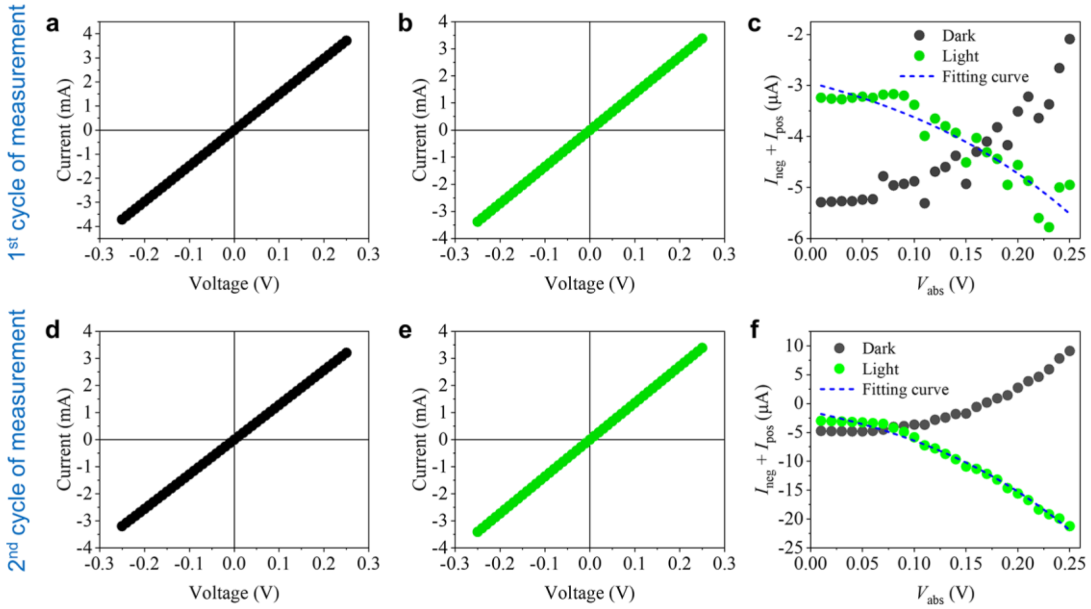

Supplementary Figure 22. In-plane electric characterization of the vertical ITO/$PEA_2(Cs_{1.33}MA_{1.33})Pb_{3.67}I_{12}$/Cu configuration. Two cycles of the *I*-*V* measurement of the vertical ITO/$PEA_2(Cs_{1.33}MA_{1.33})Pb_{3.67}I_{12}$/Cu. (a)-(c) are the first-cycle results, (d)-(f) are the second-cycle results. The *I*-*V* characteristics measured (a)&(d) in darkness and (b)&(e) under one-sun illumination. (c)&(f) (I_{neg} + I_{pos}) versus V_{abs} characteristics in darkness and under illumination.

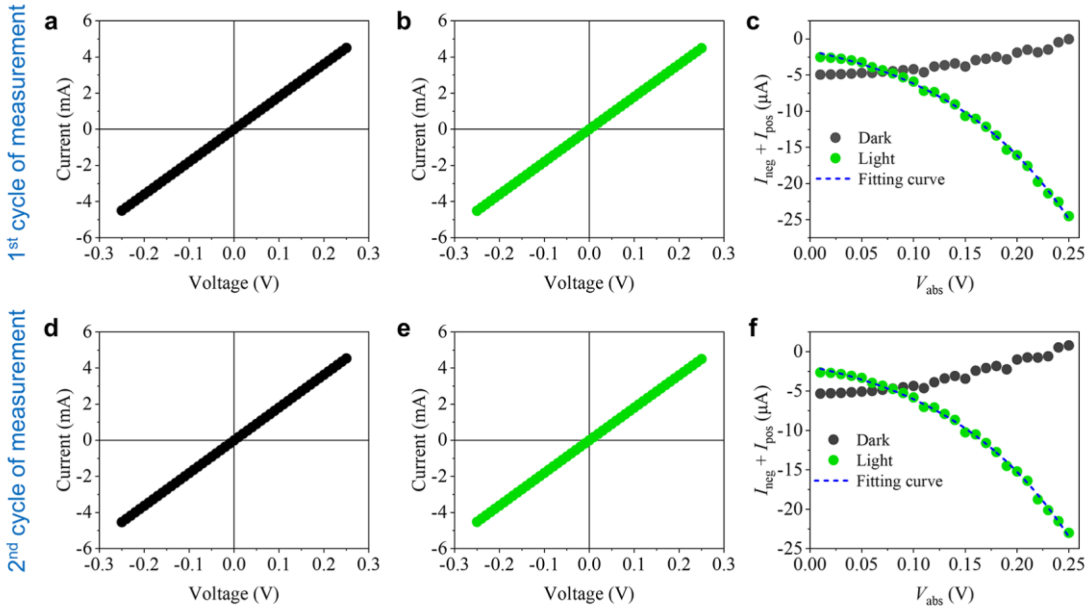

Supplementary Figure 23. In-plane electric characterization of the vertical ITO/$PEA_2(FA_{1.33}MA_{1.33})Pb_{3.67}I_{12}$/Cu configuration. Two cycles of the *I*-*V* measurement of the vertical ITO/$PEA_2(FA_{1.33}MA_{1.33})Pb_{3.67}I_{12}$/Cu. (a)-(c) are the first-cycle results, (d)-(f) are the second-cycle results. The *I*-*V* characteristics measured (a)&(d) in darkness and (b)&(e) under one-sun illumination. (c)&(f) (I_{neg} + I_{pos}) versus V_{abs} characteristics in darkness and under illumination.

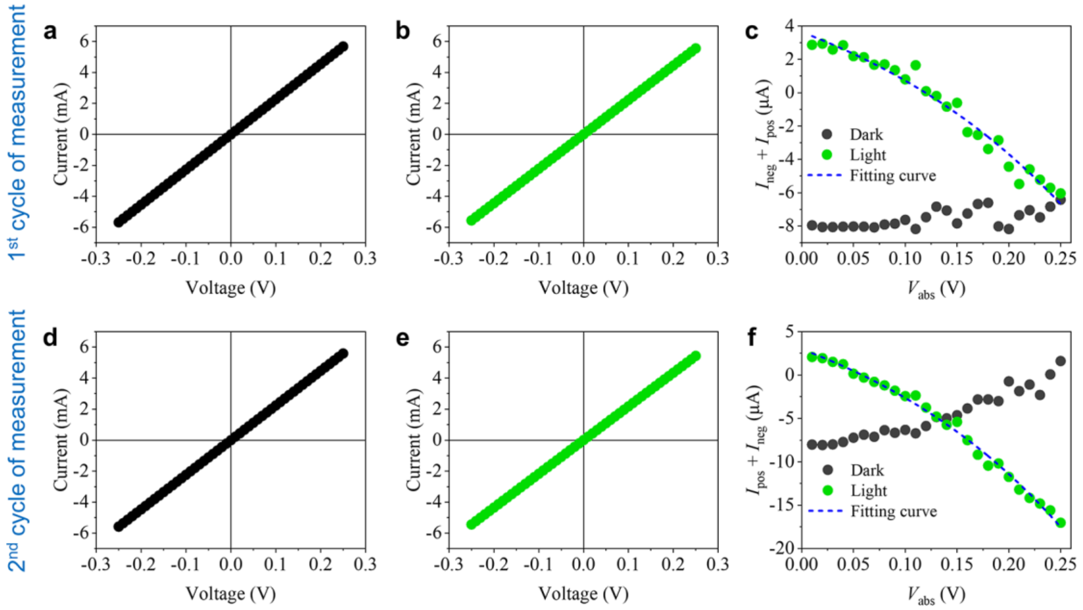

Supplementary Figure 24. In-plane electric characterization of the vertical ITO/ $PEA_2(Cs_{0.67}FA_{0.67}MA_{1.33})Pb_{3.67}I_{12}$/Cu configuration. Two cycles of the *I*-*V* measurement of the vertical ITO/ $PEA_2(Cs_{0.67}FA_{0.67}MA_{1.33})Pb_{3.67}I_{12}$/Cu. (a)-(c) are the first-cycle results, (d)-(f) are the second-cycle results. The *I*-*V* characteristics measured (a)&(d) in darkness and (b)&(e) under one-sun illumination. (c)&(f) (I_{neg} + I_{pos}) versus V_{abs} characteristics in darkness and under illumination.

Supplementary Table 1. Summary of the photoinduced potential barriers in various mixed-dimensional 2D/3D HP systems

A-site cation		MA (n= 3.67)	$Cs_{0.5}MA_{0.5}$ (n= 3.67)	$FA_{0.5}MA_{0.5}$ (n= 3.67)	$MA_{0.5}Cs_{0.25}FA_{0.25}$ (n= 4)
Photoinduced barrier (meV)	1st cycle	165.5	204.5	126.8	242.0
	2st cycle	91.6	176.8	125.5	224.1

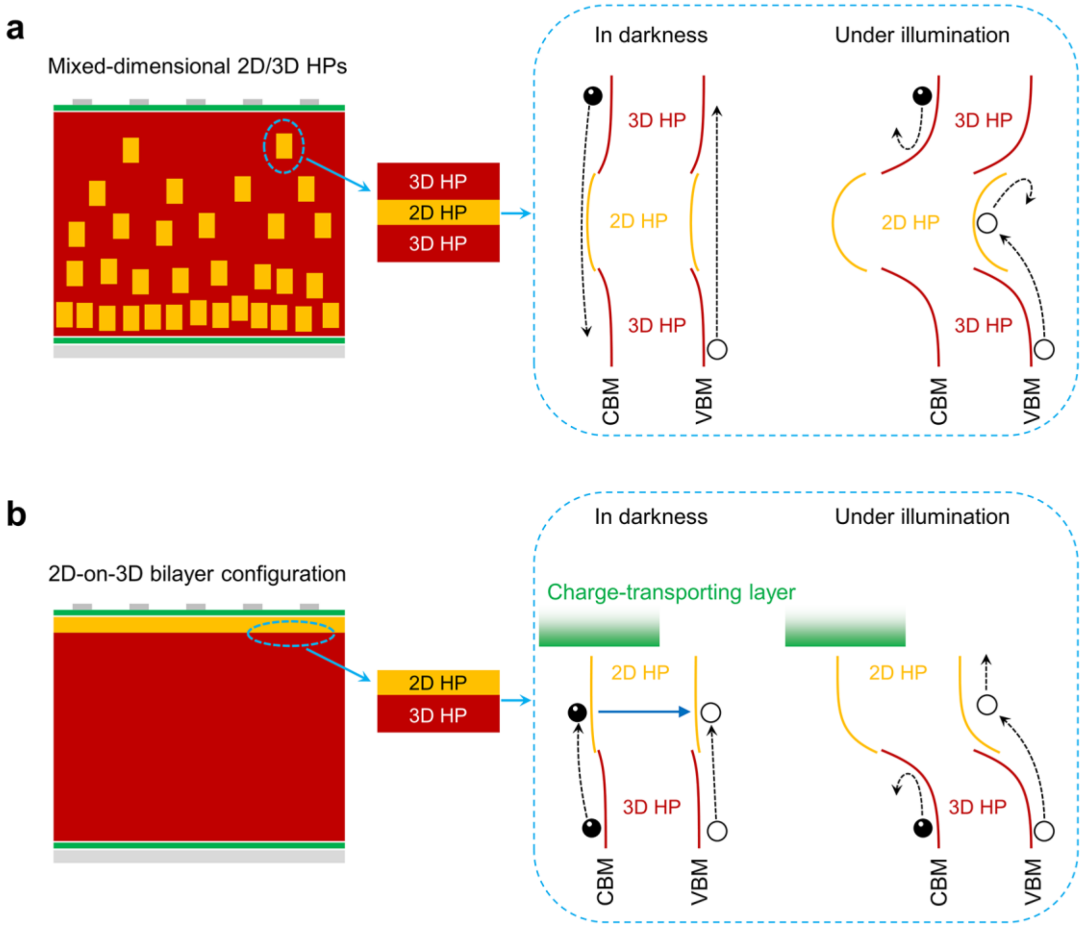

Supplementary Figure 25. The difference between the mixed-dimensional 2D/3D configuration and the 2D-on-3D bilayer configuration. The effect of light-enhanced built-in potential of the 2D/3D HP interface in (a) the mixed-dimensional 2D/3D HP solar cells and (b) the 2D-on-3D HP solar cells. The black circles represent electrons, and the white circles represent holes.

Supplementary Note 7: While the light-enhanced built-in potential would also occur in the 2D-on-3D bilayer configuration the same as in the mixed-dimensional 2D/3D configuration, it would not lead to the PCB effect. Such a difference arises from the different phase arrangements of the two systems. In the mixed-dimensional 2D/3D system, 2D HP fragments intersperse in the 3D HP matrix, thus giving rise to a local 3D/2D/3D arrangement (Supplementary Figure 25a). Upon illumination, the built-in potential of the 2D/3D HP interface increases, and electrons that otherwise could freely transport through the local 3D/2D/3D site would be inhibited by the 2D HP fragment. In a similar way, holes would be deeply trapped by the 2D HP fragment.

However, for the 2D-on-3D bilayer system, there is no such local 3D/2D/3D arrangement. The 2D HP layer caps the top surface of the 3D HP layer to serve as not only a passivating layer but also a carrier transporting layer. Carriers generated in the 3D HP layer would pass through the top 2D HP layer before being collected by the real charge transporting layer (Supplementary Figure 25b). As such, the light-enhanced built-in potential of the 2D/3D interface would, in turn, promote carrier harvesting as it facilitates carrier separation to suppress the lossy recombination.

Supplementary References

1. Yu, D. et al. Broadband and sensitive two-dimensional halide perovskite photodetector for full-spectrum underwater optical communication. *Nano Research* **14,** 1210-1217 (2020).
2. Lin, Y. et al. Suppressed ion migration in low-dimensional perovskites. *ACS Energy Lett.* **2,** 1571-1572 (2017).
